# Rapid and Sensitive Detection of *Candida albicans* Using Microfluidic-Free Droplet Digital Non-Amplification Dependent CRISPR/Cas12a Assay

**DOI:** 10.3390/bios16020072

**Published:** 2026-01-26

**Authors:** Jie Peng, Chao Guo, Ze-Yun Huang, Wen-Fei Xu, Xu-Hui Li

**Affiliations:** 1 Women’s Hospital, School of Medicine, Zhejiang University, Hangzhou 310006, China; 2 Zhejiang Key Laboratory of Multiomics and Molecular Enzymology, Yangtze Delta Region Institute of Tsinghua University, Zhejiang, Jiaxing 314006, China; 3 College of Fisheries and Life Science, Shanghai Ocean University, Shanghai 201306, China

**Keywords:** digital PCR, CRISPR/Cas12a, candida albicans detection

## Abstract

*Candida albicans* is a major fungal pathogen associated with vulvovaginal candidiasis, and rapid, sensitive detection remains challenging, particularly in amplification-free formats. Here, we report NaPddCas, a microfluidic-free, droplet-based CRISPR/Cas12a detection strategy for qualitative identification of *Candida albicans* DNA. Unlike conventional bulk CRISPR assays, NaPddCas partitions the reaction mixture into vortex-generated polydisperse droplets, enabling spatial confinement of Cas12a activation events and effective suppression of background fluorescence. This compartmentalization substantially enhances detection sensitivity without nucleic acid amplification or microfluidic devices. Using plasmid and genomic DNA templates, NaPddCas achieved reliable detection at concentrations several orders of magnitude lower than bulk CRISPR/Cas12a reactions. The assay further demonstrated high specificity against non-target bacterial and fungal species and was successfully applied to clinical vaginal secretion samples. Importantly, NaPddCas is designed as a qualitative or semi-qualitative droplet-dependent digital detection method rather than a quantitative digital assay. Owing to its simplicity, sensitivity, and amplification-free workflow, NaPddCas represents a practical approach for laboratory-based screening of *Candida albicans* infections.

## 1. Introduction

*Candida albicans* is a major fungal pathogen that significantly affects women’s health, serving as the primary pathogenic factor of vulvovaginal candidiasis (VVC) [1]. This opportunistic infection can severely impair reproductive health and is associated with pregnancy complications such as premature rupture of membranes, preterm labor, chorioamnionitis, and congenital cutaneous candidiasis [2]. Notably, approximately 75% of women experience at least one episode of symptomatic VVC during their lifetime, and about 10% develop recurrent infections [3], highlighting its widespread clinical burden. Despite advances in antifungal therapeutics, including broader drug classes and reduced toxicity [4], recent epidemiological studies suggest that these treatments have had limited effect in decreasing VVC prevalence [5]. One of the main obstacles to effective management is the diagnostic challenge. Clinical manifestations of VVC are often nonspecific and difficult to distinguish from bacterial infections. As a result, antifungal therapy is frequently empirical and delayed, especially when resistance to antibiotics prompts suspicion of fungal etiology. Current diagnostic methods such as microscopic examination and culture of vaginal secretions-suffer from low specificity and sensitivity, respectively [6]. Therefore, there is an urgent need for the development of rapid and accurate diagnostic strategies, especially in resource-limited settings where delayed diagnosis exacerbates antifungal misuse and resistance.

CRISPR (Clustered Regularly Interspaced Short Palindromic Repeats) and its associated Cas proteins have emerged as powerful tools for nucleic acid detection [7]. In particular, CRISPR/Cas12a, an endonuclease guided by crRNA, exhibits both cis-cleavage of specific double-stranded or single-stranded DNA and trans-cleavage of non-target single-stranded DNA, enabling signal amplification through reporter cleavage [8,9]. This property has facilitated the development of fluorescence-based detection methods such as DETECTR [8] and HOLMES [9]. However, these assays often require multiple handling steps and nucleic acid amplification, which increases the risk of contamination [10]. Subsequent methods such as STOPCovid.v1 and SHINE have aimed to simplify workflows, but their sensitivity is lower than quantitative PCR (qPCR) [11,12]. Recent advancements in digital CRISPR technologies have improved detection sensitivity [13,14,15], but their reliance on sophisticated microfluidic platforms and target amplification has hindered clinical application. Therefore, there remains an unmet need for user-friendly CRISPR-based diagnostics that achieve high sensitivity and specificity without complex equipment and procedures.

Recent studies have explored amplification-free CRISPR-based nucleic acid detection to simplify workflows and reduce contamination risk; however, these approaches typically suffer from limited sensitivity in bulk reactions [16]. Digital CRISPR strategies have been introduced to improve sensitivity by reaction partitioning, but most reported systems rely on monodisperse microfluidic droplets and upstream nucleic acid amplification [17]. Polydisperse droplet systems generated by simple emulsification methods have been less explored in CRISPR diagnostics, particularly in amplification-free formats. Therefore, a simple, amplification-free CRISPR detection strategy that leverages droplet compartmentalization without microfluidics remains underdeveloped.

To address these limitations, we developed a non-amplification-assisted CRISPR/Cas12a assay integrated with a polydisperse droplet digital system (NaPddCas) for the sensitive and specific detection of *Candida albicans* DNA. This assay can detect as low as 10 copies/μL of target DNA within 30 min, requiring only basic instrumentation and no microfluidic devices. We further confirmed the method using clinical vaginal samples, demonstrating high sensitivity and specificity. Owing to its simplicity, cost-effectiveness, and diagnostic accuracy, NaPddCas holds great promise as a useful diagnostic strategy for vaginal *Candida albicans* infections, particularly in low-resource environments.

## 2. Materials and Methods

### 2.1. Chemicals and Reagents

CRISPR RNA (crRNA) was synthesized by Bio-life Sci (Guangzhou, China), and LbaCas12a was purchased from New England Biolabs (Beijing, China). DNA/RNase-free water and ssDNA-FQ reporter were obtained from Sangon Biotech (Shanghai, China). Bovine serum albumin (BSA) was purchased from Macklin (Shanghai, China). Plasmids containing the ITS1/2 region of *Candida albicans* genomic DNA (gDNA) and other chemical reagents were sourced from Sangon Biotech. The 24-well plate was purchased from Shanghai Jingan Biotech (Shanghai, China), and the yeast gDNA extraction kit was obtained from Tiangen Biotech (Beijing, China). Genomic DNA of *Candida albicans*, *Pseudomonas aeruginosa*, *Staphylococcus aureus*, *Acinetobacter baumannii*, *Escherichia coli*, and *Candida glabrata* were purchased from Bena culture collection (Beijing, China). All oligonucleotides used are listed in Table S1.

### 2.2. Bulk CRISPR-Cas12 Activation Assay

Each 12.5 μL CRISPR-Cas12a reaction contained 100 nM LbaCas12a, 100 nM crRNA, 500 nM ssDNA-FQ reporter, 1× NEBuffer™ r2.1 (10 mM Tris-HCl, 50 mM NaCl, 10 mM Mg^2+^, 100 μg/mL recombinant albumin), and target DNA plasmids. Reactions were incubated at 37 °C for up to 60 min using a Real-Time PCR System (ABI Q3, Applied Biosystems, Carlsbad, CA, USA), with fluorescence recorded every minute.

### 2.3. Optimization of Reaction Parameters

To optimize CRISPR-Cas12a activity, real-time fluorescence assays were performed using different temperatures (37 °C, 39 °C, 41 °C, 43 °C, and 45 °C). The reaction mixture included 100 nM Cas12a, 100 nM crRNA, 500 nM reporter, and plasmid DNA. The Mg^2+^ concentrations (0–35 mM) and BSA concentrations (0–6 mg/mL) were applied to determine optimal conditions. Subsequently, different Cas12a concentrations (0–200 nM) and crRNA: Cas12a ratios (1:1 to 1:2) were tested under optimized conditions. All assays were performed on the ABI Q3 platform.

### 2.4. Droplet Digital Detection of Candida albicans DNA

A 3 μL reaction mixture contained 150 nM Cas12a, 195 nM crRNA, 500 nM ssDNA-FQ reporter, 25 mM Mg^2+^, 4 mg/mL bovine serum albumin, 1× optimized reaction buffer, and the indicated amount of target DNA template, was emulsified in 100 μL of oil (90% isopropyl palmitate, 10% Abil EM 180, *v*/*v*) by vortex mixing to generate polydisperse droplets. Following incubation at 41 °C for 30 min, the droplets were uniformly distributed onto a glass slide. Fluorescence images (4 mm × 4 mm) were subsequently captured using a Nikon Eclipse Ti2 inverted fluorescence microscope (Nikon Corporation, Tokyo, Japan) equipped with a 20× objective lens to acquire comprehensive fluorescence signals from all droplets.

### 2.5. Specificity Assessment of NaPddCas Assay

Genomic DNA from *Candida albicans* and non-target organisms including *Pseudomonas aeruginosa*, *Staphylococcus aureus*, *Acinetobacter baumannii*, *Escherichia coli*, and *Candida glabrata* was used to conduct the NaPddCas assays. The number of positive droplets was counted to evaluate the specificity of NaPddCas for *Candida albicans* detection.

### 2.6. Clinical Sample Analysis

Clinical vaginal secretion samples were collected from the Second Affiliated Hospital of Jiaxing University (Zhejiang, Jiaxing, China), with approval from the Independent Ethics Committee of the Second Affiliated Hospital of Jiaxing University. For each clinical sample, 200 μL of vaginal secretion was used for genomic DNA extraction using a commercial kit according to the manufacturer’s protocol (Beijing, Tiangen Biotech). DNA was eluted in 50 μL elution buffer, of which 1.25 μL was added to the NaPddCas reaction mixture, and then tested with the NaPddCas assay. After a 30 min reaction, 20 μL of the mixture was plated in a 24-well plate using a microplate horizontal shaker and imaged with the Nikon Eclipse Ti2 system under standardized parameters (exposure: 100 ms; gain: 5.1×; objective: 20×).

### 2.7. Data Processing and Statistical Analysis

Data processing and statistical analysis were conducted using Image J software (version 1.47). The threshold for identifying positive droplets was established as the average fluorescence intensity of the negative control (NTC) plus three times its standard deviation. To quantify the total number of positive droplets, the original image was initially transformed into an eight-bit format. Subsequently, a threshold was applied to convert the image into a binary representation, thereby eliminating negative droplets. Finally, the “Analyze Particles” function was employed to enumerate the positive droplets. Statistical analyses were carried out using GraphPad Prism software (version 8.0.1).

For clinical classification, a sample was considered positive if its droplet count exceeded the mean of negative samples (patients without *Candida albicans* infection) plus three standard deviations. Statistical analysis was performed using GraphPad Prism (version 8.0.1). All experiments were repeated at least three times. Two-tailed Student’s *t*-tests were used to determine significance.

## 3. Results and Discussion

### 3.1. Principle of NaPddCas Assay

**Scheme 1:**
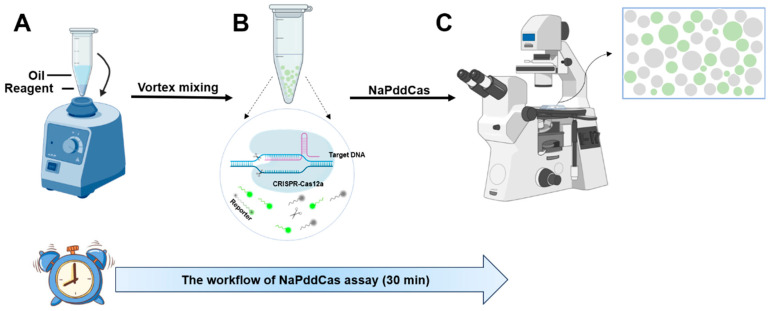
Schematic illustration of the NaPddCas assay for the detection of *Candida albicans* DNA. (**A**) The reagent mixture and oil were vortexed to create polydisperse emulsions. (**B**) The target DNA mediated the activation of Cas12a in droplets to cleave the FAM-modified ssDNA to generate green fluorescence. (**C**) The droplets with fluorescence signals were detected by the fluorescence microscope.

The principle of NaPddCas for *Candida albicans* detection is illustrated in Scheme 1. This approach integrates polydisperse droplets with CRISPR/Cas12a biosensors to enable digital detection of *Candida albicans* DNA. Briefly, the reaction mixture, consisting of the Cas12a system and oil, was combined at a 1:10 ratio, and polydisperse droplets were generated by vortex mixing. Subsequently, the CRISPR/Cas12a system specifically recognized target DNA, activating the trans-cleavage activity of Cas12a. This activation led to the indiscriminate cleavage of ssDNA reporters, releasing fluorescence signals. The fluorescence-positive microdroplets were then visualized using a fluorescence microscope, whereas non-target nucleic acids failed to induce Cas12a activation. This strategy enables the detection of *Candida albicans* with high sensitivity and specificity.

### 3.2. Feasibility of the NaPddCas Assay

**Figure 1:**
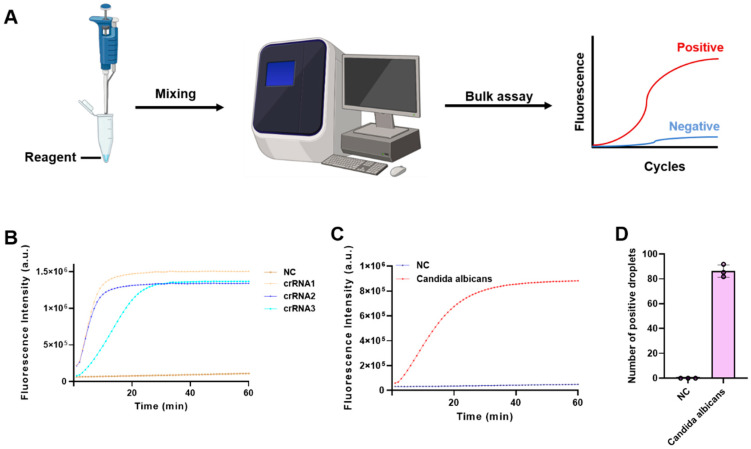
Feasibility of the bulk fluorescence Cas12a assay and NaPddCas for *Candida albicans* DNA detection. (**A**) Schematic of the bulk fluorescent Cas12a assay for *Candida albicans* DNA detection. (**B**) Real-time fluorescence curves of the Cas12a activation assay mediated by three different crRNAs for the *Candida albicans* DNA detection. NC means the negative control (water as template). (**C**) Real-time fluorescence curves of the bulk Cas12a activation assay for *Candida albicans* DNA detection. NC means the negative control (water as template). (**D**) Number of positive droplets of the NaPddCas assay for *Candida albicans* DNA detection. NC refers to the negative control (water as template).

A fluorescent bulk Cas12a activation assay was first conducted to verify the collateral cleavage activity of Cas12a, which serves as the biochemical basis for subsequent NaPddCas development (Figure 1A). Three crRNA sequences targeting the ITS1/2 region of *Candida albicans* were designed for CRISPR/Cas12a activation (Table S1). All crRNA-containing groups exhibited obvious fluorescence signals, whereas the control group (without crRNA) did not generate any detectable signal when using *Candida albicans* gDNA as the template (Figure 1B). Among the tested crRNAs, crRNA1 targeting IS2 gene of *Candida albicans* exhibited the strongest fluorescence signal and the shortest time to reach plateau (Figure 1B), leading to its selection for subsequent assays. To further explore potential factors contributing to the superior performance of crRNA1, in silico secondary structure analysis was performed for all three crRNAs. RNA folding prediction suggested that crRNA1 exhibits a more accessible spacer region with reduced intramolecular base pairing compared with crRNA2 and crRNA3 (Figure S1 and Table S2). Such structural features have been reported to influence Cas12a activation efficiency by facilitating target recognition and R-loop formation [18,19,20]. It should be noted that this analysis is predictive in nature, and the observed correlation does not imply direct mechanistic validation.

To further assess feasibility, a Cas12a activation assay was performed using plasmids containing the IS2 gene of *Candida albicans* as the template. The results confirmed fluorescence signals in the *Candida albicans* group, with no detectable signals in the control group (Figure 1C). Classical CRISPR-based diagnostic platforms require nucleic acid reamplification or microfluidic chips to improve detection sensitivity [10,21]. However, this requirement complicates procedures and increases the risk of aerosol-mediated contamination during disease diagnosis. To overcome these limitations, the NaPddCas assay was developed, integrating Cas12a activation within polydisperse droplets. As shown in Figure 1D and Figure S2, the number of fluorescence-positive droplets was significantly higher in the *Candida albicans* group compared to the control group, indicating the application potential of the NaPddCas assay for *Candida albicans* detection.

### 3.3. Optimization

**Figure 2:**
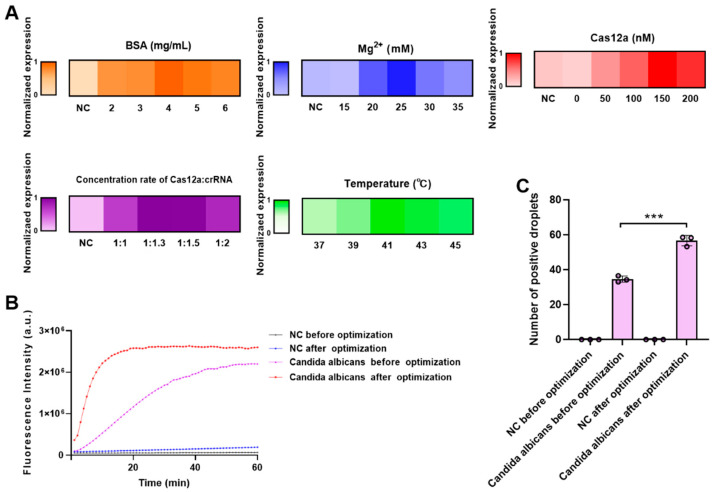
Optimization of the NaPddCas system. (**A**) Optimization of the reaction temperature and buffer compositions for the CRISPR-Cas12a assay. The darkest color indicates the optimum condition. (**B**) Real-time fluorescence curves of the bulk Cas12a activation assay for *Candida albicans* DNA detection before and after optimization. NC refers to the negative control (water as template). (**C**) Number of positive droplets of the NaPddCas assay for *Candida albicans* DNA detection before and after optimization. NC refers to the negative control (water as template). (*** *p* < 0.001).

A series of fluorescent Cas12a assays were performed to optimize key parameters of the Cas12a activation system and enhance its sensitivity for nucleic acid detection. Reaction conditions, including temperature, buffer composition, and the concentrations of Mg^2+^, BSA, Cas12a, and crRNA-to-Cas12a ratios, were evaluated (Figure 2A). Real-time fluorescence analysis demonstrated enhanced Cas12a activation following optimization (Figure 2B). Subsequently, the feasibility of the optimized Cas12a activation assay was assessed in polydisperse droplets. The results showed a greater number of fluorescence-positive droplets under optimized conditions compared to the initial experimental conditions (Figure 2C). These results indicate that the optimized NaPddCas system could be applied to detect *Candida albicans* DNA in a droplet-based manner.

### 3.4. Performance of the NaPddCas Assay for Candida albicans DNA Detection

**Figure 3:**
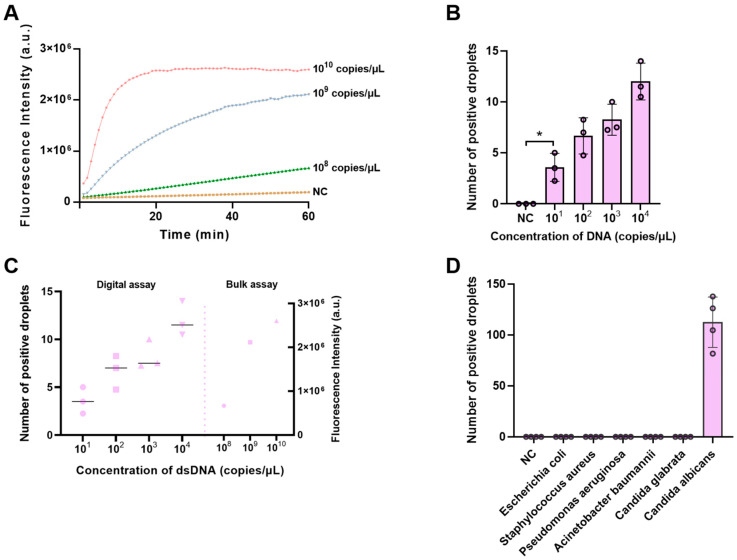
Performance analysis of NaPddCas system for nucleic acids detection. (**A**) Real-time fluorescence curves of the bulk Cas12a activation assay for *Candida albicans* DNA detection at different concentrations. NC refers to the negative control (water as template). (**B**) Number of positive droplets of NaPddCas assay at different concentrations of plasmid containing *Candida albicans* DNA. NC refers to the negative control (water as template). (* *p* < 0.05). (**C**) Comparison of the LOD of the NaPddCas assay and the corresponding bulk CRISPR-Cas12 activation assay. (**D**) The specificity of NaPddCas assay was tested by using the genomic DNA of common microorganisms causing vaginitis (*Pseudomonas aeruginosa*, *Staphylococcus aureus*, *Acinetobacter baumannii*, *Escherichia coli*, *Candida glabrata* and *Candida albicans*).

To evaluate the sensitivity of the NaPddCas system, a series of diluted plasmids containing *Candida albicans* DNA were tested under optimized conditions. In bulk reactions, fluorescence signals were indistinguishable from the no-template control at low target concentrations, indicating limited sensitivity in non-compartmentalized formats. As shown in Figure 3A, the fluorescence signal intensity increased with the increase in DNA concentrations. The real-time Cas12a activation assay can detect as few as 1 × 10^8^ copies/μL of *Candida albicans* DNA after optimization (Figure 3A). Furthermore, fluorescence microscopy was used to analyze droplet signals at different reaction times, with positive droplets counted after incubation for 0–60 min. The results indicated clear differentiation between target DNA-containing groups and the control group as early as 15 min, with the number of positive droplets peaking at 30 min (Figure S3). A greater number of fluorescence-positive droplets were observed in samples with the increase in target DNA concentrations (Figure 3B and Figure S4). Moreover, NaPddCas generated discrete fluorescent droplets at substantially lower target concentrations, enabling reliable discrimination from negative controls. The limit of detection (LOD) of NaPddCas was defined as the lowest target concentration that produced a fluorescence signal exceeding the mean background signal plus three standard deviations of the no-template control. Importantly, due to the polydisperse nature of vortex-generated droplets, the LOD reflects qualitative detectability rather than absolute quantification. Notably, as low as 10 copies/μL of *Candida albicans* DNA was detected by the NaPddCas assay (Figure 3B and Figure S4), demonstrating a sensitivity about 10^7^-fold higher than that of the real-time bulk assay (Figure 3C). Importantly, the enhanced sensitivity of NaPddCas does not rely on treating droplets as independent statistical replicates. Instead, droplet compartmentalization spatially confines CRISPR activation events, suppresses background fluorescence, and enables localized signal accumulation, thereby improving detectability of low-abundance targets compared with bulk reactions. Notably, due to the polydisperse nature of vortex-generated droplets, NaPddCas is not intended for Poisson-based absolute quantification. Moreover, because droplets generated by vortex emulsification are inherently polydisperse, droplets of different volumes may contribute unequally to the observed fluorescence signals. In the NaPddCas assay, this polydispersity is not corrected or normalized for absolute quantification. Instead, the analysis is intentionally based on droplet-level qualitative classification (fluorescence-positive versus negative) rather than volume-weighted signal integration. Larger droplets may produce stronger fluorescence signals due to higher reporter content; however, this effect is leveraged to enhance qualitative detectability rather than treated as a source of analytical bias.

To assess the specificity for *Candida albicans* detection, the NaPddCas assay was performed using both target and non-target DNA as the templates. Specifically, *Candida albicans* gDNA served as the target, while gDNA from *Pseudomonas aeruginosa*, *Staphylococcus aureus*, *Acinetobacter baumannii*, *Escherichia coli*, and *Candida glabrata* were used as non-target controls. The results demonstrated that non-target DNA did not generate detectable signals (Figure 3D and Figure S5). It should be noted that the analytical LOD established here was determined using defined DNA templates under controlled conditions and does not represent the clinical limit of detection in vaginal samples, where additional pre-analytical and biological factors may influence assay performance. Together, these findings demonstrate that the NaPddCas system enables rapid, sensitive, and highly specific detection of *Candida albicans*.

### 3.5. Detection of Candida albicans in Clinical Samples Using the NaPddCas Assay

**Figure 4:**
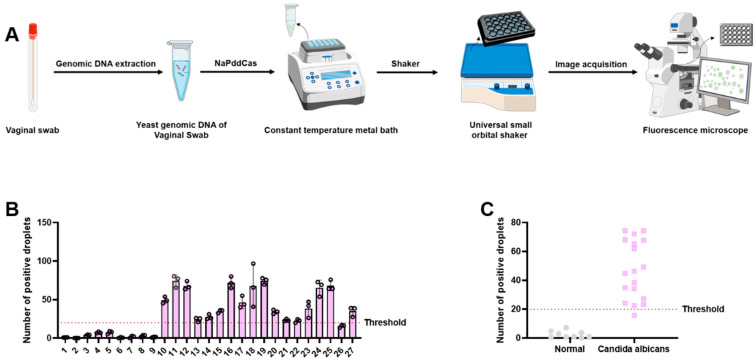
Clinical validation of NaPddCas system. (**A**) Schematic of NaPddCas for *Candida albicans* DNA detection in clinical samples. (**B**,**C**) Clinical samples test results for 27 vaginal swab samples detected by the NaPddCas assay (left, 9 negative samples (patients without *Candida albicans* infection); right, 18 positive samples (patients with *Candida albicans* infection)). The red line represents the detection threshold The threshold was set as 20 (average number of droplets of the negative samples plus its triple-variance).

To standardize detection processing, we first established a microplate orbital shaker-assisted droplet plating system. Using 24-well glass-bottom plates, we evaluated three critical parameters: shake rotation speed, plating volume, and shake duration. As shown in Figure S6, the optimal condition was achieved at 600 rpm with a plating volume of 20 μL and a shaking time of 120 s. Then, we evaluated the performance of the NaPddCas assay under the standardized photography parameters (exposure: 100 ms; gain: 5.1×). The results demonstrated that obvious fluorescent droplets were detected in positive samples but not in the negative controls (Figure S7), indicating the feasibility of high-throughput detection of *Candida albicans* using the NaPddCas system. For clinical sample analysis, a positivity threshold was defined based on the number of positive droplets rather than absolute fluorescence intensity. Specifically, the threshold (20 droplets) was calculated as the mean positive droplet count of negative control samples (mean = 2 droplets) plus three times the corresponding standard deviation (SD = 6 droplets), a commonly used criterion in droplet-based fluorescence assays to distinguish background-level signals from true positive events. This threshold was applied exclusively for qualitative classification rather than absolute quantification. To evaluate the clinical applicability of the NaPddCas assay, we tested gDNA extracted from vaginal swabs of patients (Table S3). As shown in Figure 4B,C, the NaPddCas system successfully correctly classified 17 out of 18 positive clinical samples (17/18) and correctly identified all negative samples (9/9) as non-*Candida albicans* based on positive droplet counts below the predefined positivity threshold. Thus, the assay demonstrated a sensitivity of 94.4% and a specificity of 100% for *Candida albicans* detection (Table S4).

## 4. Conclusions

In this study, we developed a novel digital detection strategy, named “NaPddCas”, for the diagnosis of *Candida albicans* infection. The NaPddCas assay offers several advantages: (1) High sensitivity and specificity: By integrating polydisperse droplet technology with the CRISPR/Cas12a system, NaPddCas achieves excellent sensitivity and specificity. Under optimized conditions, the assay detects *Candida albicans* DNA at a limit of 10 copies/μL, comparable to conventional qPCR-based methods [22]. (2) Rapid and cost-effective: NaPddCas offers a simplified workflow compared to traditional digital PCR, requiring only a vortex mixer, a thermostat, a microplate horizontal shaker, and a fluorescence microscope, which eliminates the need for microfluidic chips. The equipment cost remains under $1000, and the per-reaction cost does not exceed $10. Moreover, the assay’s cost-effectiveness can be further improved by substituting the fluorescence microscope with a smartphone-integrated portable FAM-channel testing system. (3) Potential for point-of-care testing (POCT): The unique combination of polydisperse droplet-dependent CRISPR/Cas12a and a portable FAM-channel test system integrated into a smartphone enables the NaPddCas assay to serve as a promising POCT platform for the detection of *Candida albicans*.

We demonstrate that NaPddCas substantially improves the detectability of *Candida albicans* DNA compared with bulk CRISPR/Cas12a reactions. Importantly, this enhancement should not be interpreted as Poisson-based digital quantification. Unlike conventional droplet digital PCR or microfluidic digital CRISPR platforms, the droplets in NaPddCas are generated by vortex emulsification and are intentionally polydisperse. Therefore, individual droplets cannot be treated as statistically independent replicates, and absolute target copy number estimation is not the aim of this method. Instead, NaPddCas functions as a droplet-dependent digital detection strategy, in which spatial compartmentalization confines Cas12a activation events, reduces background fluorescence accumulation, and enables localized signal enrichment. This compartmentalization effect increases the signal-to-background ratio, allowing low-abundance targets that are undetectable in bulk reactions to be qualitatively identified. Notably, the use of vortex-generated polydisperse droplets is an intentional design choice in NaPddCas, where droplets are not treated as independent statistical replicates but as confined reaction compartments for qualitative signal discrimination.

The improved performance of NaPddCas arises from several synergistic factors associated with droplet compartmentalization. First, partitioning distributes target molecules and CRISPR reagents into confined microenvironments, which limits dilution effects present in bulk assays. Second, background reporter cleavage caused by nonspecific or basal Cas12a activity is spatially restricted, preventing global fluorescence accumulation that masks weak true-positive signals. Third, larger droplets generated during vortex emulsification may contribute disproportionately to detectable fluorescence; however, rather than being a limitation, this property is leveraged here to enhance qualitative detection sensitivity. Accordingly, droplet size heterogeneity is not corrected or normalized in the NaPddCas workflow, as the method is not designed for volume-weighted quantification but for robust qualitative detection under minimal instrumentation conditions. As a result, NaPddCas should be regarded as a qualitative or semi-qualitative detection technique designed for rapid identification of low-level targets, rather than a quantitative digital assay.

The NaPddCas assay represents a next-generation CRISPR-based diagnostic tool, offering ultra-sensitive detection of *Candida albicans* DNA within 30 min, making it highly suitable for clinical diagnosis of vaginitis caused by *Candida albicans* infection. Additionally, the approach can be further enhanced by utilizing alternative Cas12 family members or Class II type V effectors that do not require PAM sequences, potentially improving detection efficiency, sensitivity and universality. Exploring the compatibility of other Cas proteins with polydisperse droplets may yield even greater diagnostic performance. However, an important challenge of NaPddCas for *Candida albicans* detection is its limitation in absolute quantification. Unlike traditional digital assays that rely on monodisperse droplets and Poisson statistics, NaPddCas requires new statistical models to account for varying droplet volumes. The integration of deep-learning approaches could further enhance quantification accuracy and automate image classification, making it a powerful tool for *Candida albicans* detection.

From a practical perspective, NaPddCas offers a simple, amplification-free approach for sensitive pathogen detection using minimal instrumentation, making it attractive for laboratory-based screening applications. While the method does not provide absolute quantification and is not intended to replace microfluidic digital PCR platforms, its ability to qualitatively detect Candida albicans DNA at extremely low concentrations is highly relevant for early infection screening and confirmatory diagnostics. Therefore, NaPddCas should be interpreted as a droplet-dependent qualitative or semi-qualitative detection strategy, rather than a digital assay based on Poisson statistics or monodisperse droplet assumptions. The limitations associated with droplet polydispersity are explicitly acknowledged, and future work may explore controlled emulsification strategies to further improve assay robustness while maintaining the simplicity of the current workflow. In summary, given its excellent performance in detecting *Candida albicans* from clinical samples, the NaPddCas system holds great promise as a portable, rapid, sensitive, specific and cost-effective diagnostic tool for the diagnosis of patients with *Candida albicans* infection. With its potential for broad applicability and user-friendly operation, NaPddCas could play a crucial role in vulvovaginal candidiasis diagnostics, particularly in resource-limited settings.

## Data Availability

Data will be made available on request.

## Supplementary Materials

The following supporting information can be downloaded at: https://www.mdpi.com/article/10.3390/bios16020072/s1, Figure S1: Predicted secondary structures of crRNA1, crRNA2, and crRNA3 generated by mfold (UNAfold); Figure S2: Representative droplet images of the NaPddCas assay for Candida albicans DNA detection referring to Figure 1D; Figure S3: Representative droplet images and positive droplet number of the NaPddCas assay for Candida albicans DNA detection under different incubation time. Figure S4: Number of positive droplets of NaPddCas assay at different concentrations of plasmid containing Candida albicans DNA referring to Figure 3B. Figure S5: Number of positive droplets of NaPddCas assay for the specificity detection of Candida albicans DNA referring to Figure 3D. Figure S6: Optimization of the rotation speed, plating volume, and shake time of the microplate orbital shaker-assisted droplet plating system. Figure S7: Representative droplet images and positive droplet number of the NaPddCas assay for Candida albicans DNA detection using the 24-well glass-bottom plates under standardize detection processing. Table S1: The DNA and RNA sequences used in this work; Table S2: Comparison of predicted secondary structure features of three crRNAs used in this study. Spacer accessibility was calculated as the percentage of unpaired nucleotides within the spacer region based on mfold predictions; Table S3: Basic clinical characteristics of the 27 women from the study of NaPddCas assay for Candida albicans detection; Table S4: Concordance analysis between NaPddCas and vaginal secretion culture (the gold standard for Candida albicans detection) in the clinical vaginal secretion samples of patients.
