# Supplementary material for: Rapid and Sensitive Detection of Candida albicans Using Microfluidic-Free Droplet Digital Non-Amplification Dependent CRISPR/Cas12a Assay
Source: Biosensors (Basel). 2026 Jan 26;16(2):72. doi: 10.3390/bios16020072 (PMC12938024; doi:10.3390/bios16020072)
Supplement: Supplementary file 1 [file biosensors-16-00072-s001.zip › biosensors-4052241-supplementary.pdf]

Supplementary Information

This file includes:

Experimental section

Supplementary Figures (Supplementary Fig. 1 to 7)

Supplementary Tables (Supplementary Table 1 to 4)

Experimental section

RNA secondary structure prediction. Secondary structure prediction of crRNAs was performed using the mfold web server (UNAfold, http://www.unafold.org). All sequences were analyzed as linear RNA at 37°C under default ionic conditions (1 M NaCl, no divalent ions). Minimum free energy (MFE) structures were used for comparison. Spacer accessibility was calculated as the proportion of unpaired nucleotides within the 20-nt spacer region.

Supplementary figures

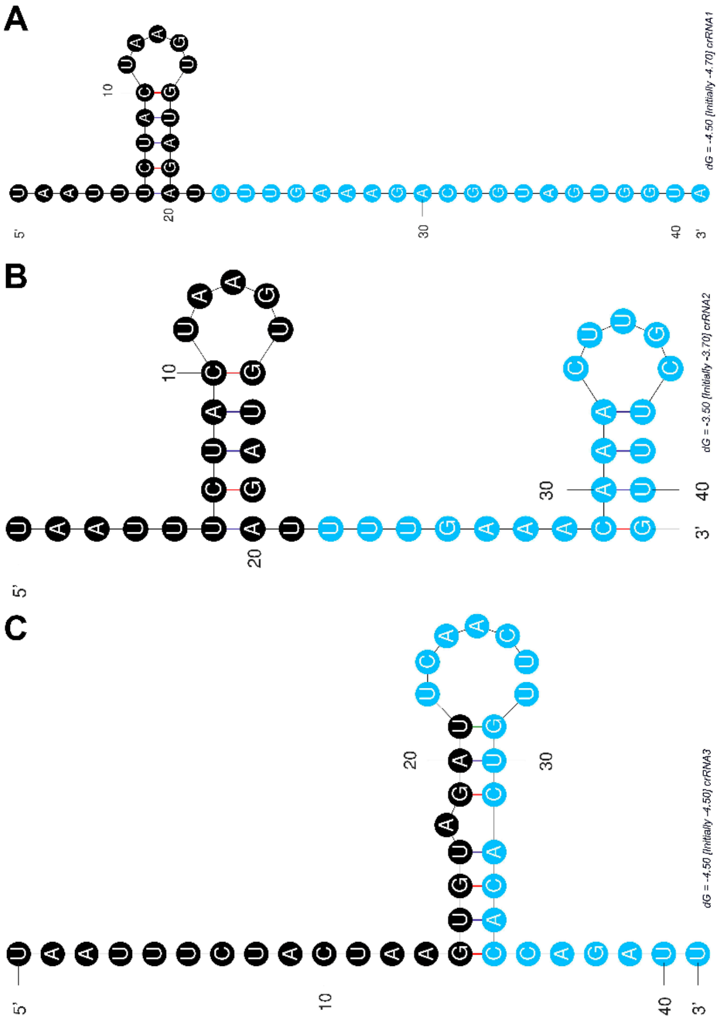

Figure S1 Predicted secondary structures of crRNA1 (A), crRNA2 (B), and crRNA3 (C) generated by mfold (UNAfold). The 5’ handle region (black background) forming the conserved stem-loop is indicated, while the spacer region (blue background) is highlighted. crRNA1 shows a largely unpaired spacer region, whereas crRNA2 and crRNA3 exhibit partial intramolecular base pairing within the spacer, potentially reducing target accessibility.

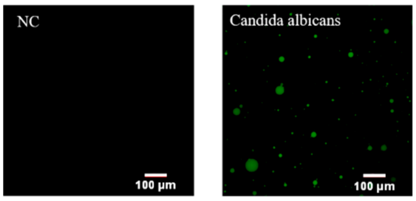

Figure S2 Representative droplet images of the NaPddCas assay for *Candida albicans* DNA detection referring to **Figure 1D**. NC refers to the negative control (water as template).

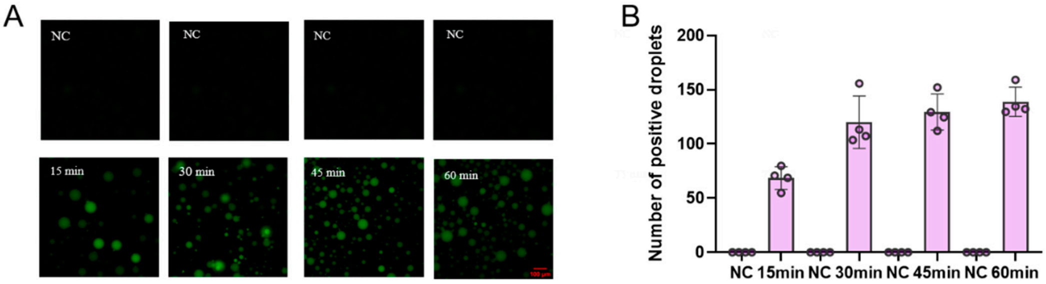

Figure S3 Representative droplet images (A) and positive droplet number (B) of the NaPddCas assay for *Candida albicans* DNA detection under different incubation time.

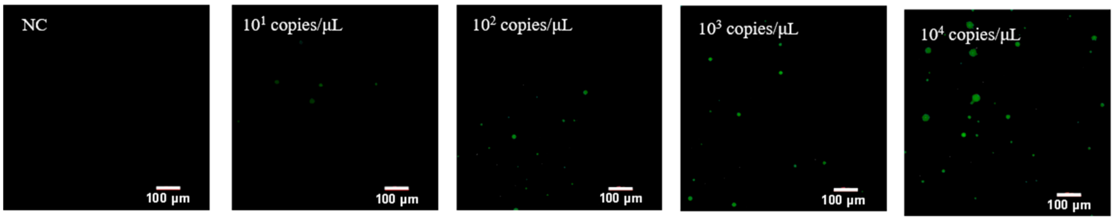

Figure S4 Number of positive droplets of NaPddCas assay at different concentrations of plasmid containing *Candida albicans* DNA referring to **Figure 3B**. NC refers to the negative control (water as template).

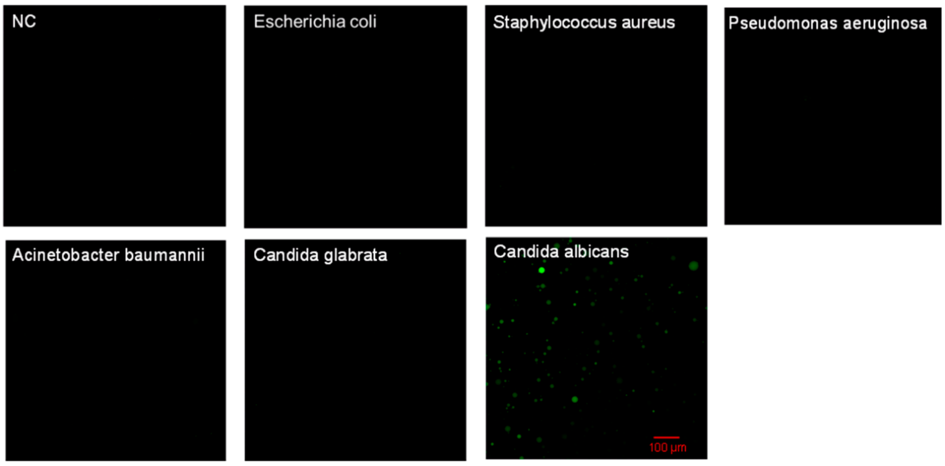

Figure S5 Number of positive droplets of NaPddCas assay for the specificity detection of *Candida albicans* DNA referring to **Figure 3D**. NC refers to the negative control (water as template).

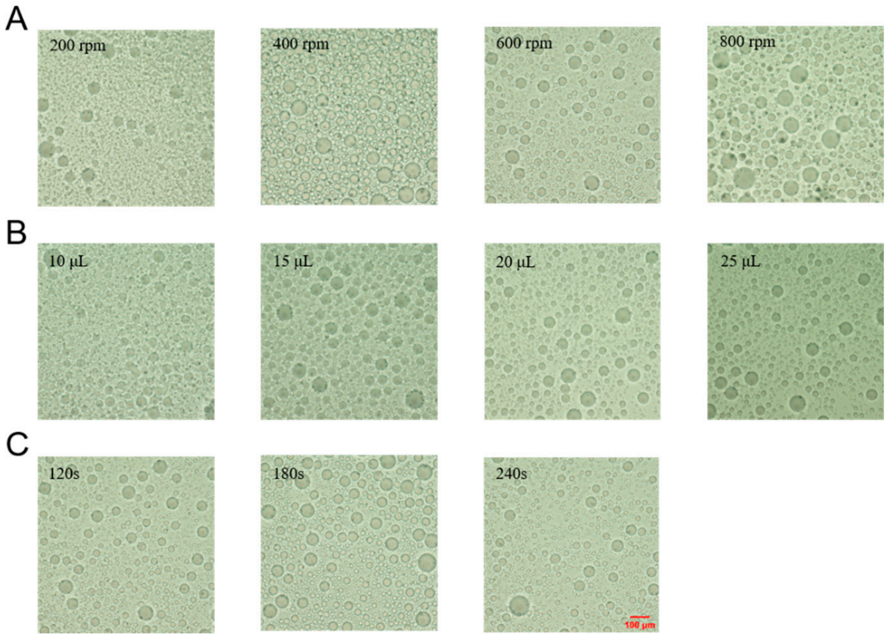

Figure S6 Optimization of the rotation speed (A), plating volume (B), and shake time (C) of the microplate orbital shaker-assisted droplet plating system.

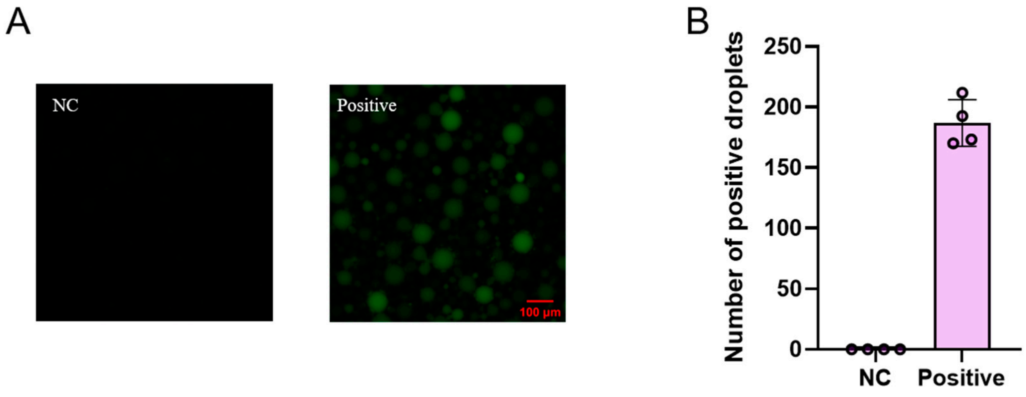

Figure S7 Representative droplet images (A) and positive droplet number (B) of the NaPddCas assay for *Candida albicans* DNA detection using the 24-well glass-bottom plates under standardize detection processing. NC refers to the negative control (water as template). Positive refers to the group using *Candida albicans* DNA as template.

Supplementary tables

Table S1 The DNA and RNA sequences used in this work

Name	Sequence (5'-3')
Target *Candida albicans* gDNA	GATCATTACTGATTTGCTTAATTGCACCACATGTGTTTTTC TTTGAAACAAACTTGCTTTGGCGGTGGGCCCAGCCTGCCG CCAGAGGTCTAAACTTACAACCAATTTTTTATCAACTTGTC ACACCAGATTATTACTAATAGTCAAAACTTTCAACAACGGA TCTCTTGGTTCTCGCATCGATGAAGAACGCAGCGAAATGC GATACGTAATATGAATTGCAGATATTCGTGAATCATCGAATC TTTGAACGCACATTGCGCCCTCTGGTATTCCGGAGGGCATG CCTGTTTGAGCGTCGTTTCTCCCTCAAACCGCTGGGTTTGG TGTTGAGCAATACGACTTGGGTTTGCTTGAAAGACGGTAG TGGTAAGGCGGGATCGCTTTGACAATGGCTTAGGTCTAACC AAAAACATTGCTTGCGGCGGTAACGTCCACCACGTATATCT TCAAACTTTGACCTCAAATCAGGTAGGACTACCCGCTGAA CTTAAGCATATCAATAAGCGGAGGAAAAGAAA
crRNA1	UAAUUUCUACUAAGUGUAGAUCUUGAAAGACGGUAGUG GUA
crRNA2	UAAUUUCUACUAAGUGUAGAUUUUGAAACAAACUUGCU UUG
crRNA3	UAAUUUCUACUAAGUGUAGAUUCAACUUGUCACACCAG AUU
Reporter DNA	6-FAM-TTTCTGTCATTC-BHQ1

Note: Yellow background: The recognition region of CRISPR/Cas12a mediated by crRNA1; Grey background: The recognition region of CRISPR/Cas12a mediated by crRNA2; Red background: The recognition region of CRISPR/Cas12a mediated by crRNA3. Blue sequence: Spacer of crRNA.

Table S2 Comparison of predicted secondary structure features of three crRNAs used in this study. Spacer accessibility was calculated as the percentage of unpaired nucleotides within the spacer region based on mfold predictions.

crRNA	MFE (kcal/mol)	Unpaired spacer bases/total	Spacer accessibility (%)	Structural feature
crRNA1	-4.7	20/20	100%	Clear stem-loop, fully exposed spacer
crRNA2	-3.7	12/20	60%	Partial spacer pairing
crRNA3	-4.5	13/20	65%	Internal pairing disrupting structure

Table S3 Basic clinical characteristics of the 27 women from the study of NaPddCas assay for *Candida albicans* detection.

Sample ID	Age（years）	VSC
1	NA	-
2	39	-
3	NA	-
4	48	-
5	29	-
6	20	-
7	40	-
8	38	-
9	31	-
10	34	+
11	46	+
12	50	+
13	34	+
14	48	+
15	22	+
16	44	+
17	40	+
18	37	+
19	20	+
20	NA	+
21	30	+
22	NA	+
23	NA	+
24	53	+
25	20	+
26	39	+
27	33	+

Note: VSC: vaginal secretion culture; NA, cases without years data; +, cases of *Candida albicans* infection; -, cases without *Candida albicans* infection.

Table S4 Concordance analysis between NaPddCas and vaginal secretion culture (the gold standard for *Candida albicans* detection) in the clinical vaginal secretion samples of patients.

		VSC		
		Positive (18)	Negative (9)	Total (27)
NaPddCas	Positive	17 (True positive)	0 (False positive)	17
	Negative	1 (False negative)	9 (True negative)	10
		Sensitivity 94.4%	Specificity 100%	

Note: VSC: vaginal secretion culture. Positive: cases of *Candida albicans* infection; Negative: cases without *Candida albicans* infection.
